# *De Novo* Transcriptome Assembly and Characterization for the Widespread and Stress-Tolerant Conifer *Platycladus orientalis*

**DOI:** 10.1371/journal.pone.0148985

**Published:** 2016-02-16

**Authors:** Xian-Ge Hu, Hui Liu, YuQing Jin, Yan-Qiang Sun, Yue Li, Wei Zhao, Yousry A. El-Kassaby, Xiao-Ru Wang, Jian-Feng Mao

**Affiliations:** 1 National Engineering Laboratory for Tree Breeding, Key Laboratory of Genetics and Breeding in Forest Trees and Ornamental Plants, Ministry of Education, College of Biological Sciences and Technology, Beijing Forestry University, Beijing, China; 2 Department of Ecology and Environmental Science, Umeå University, Umeå, Sweden; 3 Department of Forest and Conservation Sciences, Faculty of Forestry, University of British Columbia, Vancouver, British Columbia, Canada; University of Georgia, UNITED STATES

## Abstract

*Platycladus orientalis*, of the family Cupressaceae, is a widespread conifer throughout China and is extensively used for ecological reforestation, horticulture, and in medicine. Transcriptome assemblies are required for this ecologically important conifer for understanding genes underpinning adaptation and complex traits for breeding programs. To enrich the species’ genomic resources, a *de novo* transcriptome sequencing was performed using Illumina paired-end sequencing. In total, 104,073,506 high quality sequence reads (approximately 10.3 Gbp) were obtained, which were assembled into 228,948 transcripts and 148,867 unigenes that were longer than 200 nt. Quality assessment using CEGMA showed that the transcriptomes obtained were mostly complete for highly conserved core eukaryotic genes. Based on similarity searches with known proteins, 62,938 (42.28% of all unigenes), 42,158 (28.32%), and 23,179 (15.57%) had homologs in the Nr, GO, and KOG databases, 25,625 (17.21%) unigenes were mapped to 322 pathways by BLASTX comparison against the KEGG database and 1,941 unigenes involved in environmental signaling and stress response were identified. We also identified 43 putative terpene synthase (TPS) functional genes loci and compared them with TPSs from other species. Additionally, 5,296 simple sequence repeats (SSRs) were identified in 4,715 unigenes, which were assigned to 142 motif types. This is the first report of a complete transcriptome analysis of *P*. *orientalis*. These resources provide a foundation for further studies of adaptation mechanisms and molecular-based breeding programs.

## Introduction

*Platycladus orientalis* (L.) Franco, belongs to the Cupressaceae family and is a widespread and ecologically important conifer species in China [1]. It is highly adaptable and can tolerate a wide range of environmental adversities, including drought, barren soil, and mild salinity [2, 3]. Additionally, as a pioneer species, *P*. *orientalis* is often used in vegetation restoration projects in the arid mountain landscapes of northern China. *P*. *orientalis* has a unique capability: namely, absorption and accumulation of atmospheric (particulate matter, SO_2_, Cl_2_) [1] and soil pollutants (heavy metal such as Cu, Zn, among others) [4]. Therefore, it has become an important soil bioremediation tree species in many regions of China where urgent remedial actions are needed. Additionally, due to the species’ unique wood characteristics, high density and decay-resistant, it is widely used in construction, furniture, and various other industries [5, 6]. Furthermore, the species’ fruits, leaves, and bark have a long history of use in traditional Chinese medicine [7–9]. These properties, including its broad ecological range, bioremediation ability, wood quality, and medicinal applications, have led an increasing demand for effective tree improvement program targeting these traits. However, to date, knowledge of the genetic basis of these traits or of the distribution of its genetic resources is lacking.

Tree improvement activities of *P*. *orientalis* started in the 1980s and achieved success through provenance testing, clonal selection, and seed orchard establishment [6, 10, 11]. However, due to the species’ long generation time and individual size, requiring large experimental installation, conventional tree improvement methods are slow in understanding the genetic underpinning of its unique attributes and thus slowing the development of new varieties for the needed ecological restoration and environmental remediation projects. Considering the urgent need for the species germplasm conservation and utilization, a global characterization of its transcriptome is required to provide the basic genomic data for assessment and investigation of its genetic variation at the molecular level.

Modern molecular biology techniques offer novel approaches and strategies to accelerate the genetic improvement of *P*. *orientalis* through molecular breeding programs based on deciphering the molecular genetic basis of target traits. Advances in next-generation sequencing (NGS) and assembly algorithms have enabled the rapid development of next-generation RNA sequencing (RNA-Seq), which provides a rapid and cost effective way to investigate function-related transcriptome information for non-model species at low cost and with a greater sequence yield, thus simplifying the identification of functional genes, new splice variants and rare transcripts and enabling allele expression to be monitored [12, 13]. Transcriptome analysis also contributes to the development of molecular markers. Simple sequence repeats (SSRs) are important molecular markers with abundant polymorphism and are largely co-dominantly inherited, making them ideal for population genetics and molecular ecology studies. Transcriptome mining not only provides data on gene composition and expression, but also is a means of developing SSRs, thus simplifying conventional methods for developing SSR markers. RNA-Seq has been used in different plant species, from herbaceous plants to woody plants, and is particularly useful when reference is not available.

The main aim of this study was to characterize the transcriptome of *P*. *orientalis* for future gene identification, marker development and functional genomic studies of this species. We carried out *de novo* transcriptome sequencing and assembly of RNA libraries derived from terminal buds, female strobilus, biennial leaves and cambium tissues of *P*. *orientalis* adults. We provided annotation to public databases and categorized the transcripts into biological functions and pathways. In addition to analyzing the sequencing data, a set of SSR loci was also developed for future marker-based studies. This is the first report of a comprehensive characterization of the global transcriptome of *P*. *orientalis*. These new data and findings will contribute substantially to future functional genomic studies of *P*. *orientalis* and other related species.

## Materials and Methods

### Ethics Statement

All necessary permits were obtained for field studies from the Beijing Botanical Garden (Beijing, China). We also confirm that the field studies did not involve endangered or protected species.

### Plant material and RNA isolation

Terminal buds, microstrobilus (male pollen cones), female strobilus, biennial leaves, and cambium tissues were collected from five adult *P*. *orientalis* trees growing in the Beijing Botanical Garden (Beijing, China). The plant material were collected during the spring (April-May), a period of substantial plant activities. All tissues were immediately frozen in liquid nitrogen and stored at -80°C until RNA extraction. Total RNA was isolated from each tissue using an RNeasy Plant Mini Kit (Qiagen, Hilden, Germany). In total, 25 RNA samples were prepared, representing, 5 tissue-type from each of the 5 trees. These RNA samples were quantified and then mixed in equal quantities for RNA-Seq analysis.

### Construction of an mRNA-Seq library and high throughput sequencing

The mRNA library was constructed following the manufacturer’s instructions in the mRNA-Seq Sample Preparation Kit (Illumina Inc., San Diego, CA, USA). Briefly, poly-(A) mRNA was isolated from the total RNA samples using magnetic oligo (dT) beads. To avoid priming bias, the mRNA was fragmented using an RNA fragmentation kit (Ambion, Austin, TX, USA) before cDNA synthesis. Using these cleaved RNA fragments as templates, the first strand cDNA was transcribed using reverse transcriptase (Invitrogen, Carlsbad, CA, USA) and random hexamer primers. Subsequently, the second strand cDNA was synthesized using DNA polymerase I (New England BioLabs (NEB), Ipswich, MA, USA) and RNaseH (Invitrogen). The double-stranded cDNA fragments obtained were purified using T4 DNA polymerase (NEB), the Klenow fragment (NEB) and T4 polynucleotide kinase (NEB), followed by a single ‘A’ base addition using Klenow 3' to 5' exo-polymerase (NEB) to prepare the DNA fragments for ligation to the adapters, which had a single ‘T’ base overhang at the 3' end, and these were ligated using the PE Adapter Oligo Mix supplied in the mRNA-Seq Sample Preparation Kit (Illumina) using T4 DNA ligase (NEB). The products of the ligation reaction were purified according to the instructions in the MinElute PCR Purification Kit (Qiagen). The eluted adaptor-ligated fragments from the ligation reaction were separated by size on an agarose gel to select a size range of templates for downstream enrichment. The desired range of cDNA fragments (200±25 base pairs (bp)) were excised and retrieved using a Gel Extraction Kit (Axygen Biosciences, Union City, CA, USA).

Polymerase chain reaction (PCR) was performed to selectively enrich and amplify the cDNA fragments using the Phusion Master Mix (NEB) with two primers: PCR Primer PE 1.0 and PE 2.0 supplied by the mRNA-Seq Sample Preparation Kit (Illumina). These primers were annealed to the ends of the PE adapters under the following conditions: 30 sec at 98°C, then 15 cycles of 10 sec at 98°C, 30 sec at 65°C, 30 sec at 72°C, followed by 5 min at 72°C and hold at 4°C. The amplified products were purified using a QIAquick PCR Purification Kit (Qiagen). The library was prepared from a 150–200 bp size-selected fraction following adapter ligation and agarose gel separation. The library was validated for known DNA concentrations using an Eppendorf Mastercycler ep realplex Real-Time PCR System (Eppendorf, Hamburg, Germany) and sequenced using a paired end read protocol on an Illumina HiSeq^TM^ 2000 (Illumina) at Beijing Yuanquanyike Biological Technology Co., Ltd (Beijing, China).

### *De novo* transcriptome assembly

The raw reads produced following sequencing were filtered to obtain high-quality clean reads by removing the adapter sequences, ambiguous reads (reads with unknown nucleotides “N”>5%) and reads with >10% of bases with a Q-value <20. The S1 and S2 Figs shows the results of the quality assessment using FastQC [14] prior to and after trimming of poor bases and/or removal of poor reads, respectively. To assess the quality and homology of the transcriptome data, the high-quality reads of *P*. *orientalis* were mapped to the draft genomes and transcriptome assembles of *Picea abies* [15], *Picea glauca* [16, 17], *Pinus taeda* [18] by the Burrows-Wheeler alignment tool (BWA) [19]. *Picea abies*, *Picea glauca*, *Pinus taeda* are the only three published conifer species with a draft genome assembly, and all are members of the Pineceae family. Next, the transcriptome was assembled *de novo* using the Trinity short reads assembling program [20], by which clean reads with a certain length of overlap were first joined to form longer fragments, known as contigs without gaps. Subsequently, the clean reads were mapped back to contigs, with paired-end reads it is possible to detect the contigs originating from the same transcript, and also the distances between these contigs can be counted. Then, Trinity connects the contigs and obtains sequences that can no longer be extended, such sequences are defined as “unigenes”. Besides, assembled sequences of less than 200 nt were deleted.

### Identification of core eukaryotic proteins

The CEGMA pipeline [21, 22] was used to identify a subset of 248 highly conserved core eukaryotic genes (CEGs) in the resulting unigene assembly and to estimate the completeness of the core gene assembly. The CEGs were derived from 6 diverse model organisms: *Homo sapiens*, *Drosophila melanogaster*, *Arabidopsis thaliana*, *Caenorhabditis elegans*, *Saccharomyces cerevisiae*, and *Schizosaccharomyces pombe* [22].

### Assessment of gene completeness, homology to other conifer genomes and potential contamination

Gene completeness was assessed using the TRAPID (http://bioinformatics.psb.ugent.be/webtools/trapid) online tool by which all unigenes were compared against sequences in the PLAZA 2.5 green plants clade database [23]. Hits with an E-value <1.00E-5 were considered significant in the similarity search and unigenes were annotated according to the best hit values. Unigenes with one or more hits in the TRAPID database were qualified as “full-length”, “quasi full-length” or “partial” based on the length of the open reading frame (ORF). Unigenes with an ORF >2 deviations shorter than the average ORF length of the assigned gene family (excluding the 10% longest and shortest sequences within the family) were deemed as “partial”. Unigenes with an ORF longer than the mean minus 2 deviations were deemed as “full length” if they also contained a start and stop codon or as “quasi full-length” if they lacked a stop and/or start codon [24] (see S1 File for examples of how “partial”, “full” and “quasi-full” length transcript were defined).To evaluate the homology to other conifer genomes, *P*. *orientalis* unigenes were firstly mapped to the draft genome assembly of *Pinus taeda* [18] using a genomic mapping and alignment program, GMAP [25]. *P*. *orientalis* contamination were examined by blasting the unigenes without a significant hit to the PLAZA 2.5 green plants clade database to the Nr database, under the E value significance threshold of 1.00E-5, then the percentage of unigenes with significant hit to non-plant species to the total unigenes was calculated.

### Annotation and classification of the unigenes

The generated unigenes were subjected to BLASTX searches (E-value threshold of 1.00E-5) and annotated against the Non-redundant (Nr, ftp://ftp.ncbi.nih.gov/blast/db/FASTA/nr.gz), the Protein Families (Pfam, http://pfam.xfam.org/), and the Cluster of Orthologous Groups for eukaryotic complete genomes (KOG, http://genome.jgi-psf.org/help/kogbrowser.jsf) database. Additionally, Gene ontology (GO, http://www.geneontology.org) terms were extracted from the best hits obtained from the BLASTX against the Nr database using the Blast2GO program [26] with a E-value threshold of 1.00E-5, and the unigenes were assigned to molecular function, biological process, and cellular component ontologies. Next, the distribution of those unigenes’ function category was summarized using WEGO software [27]. The Kyoto Encyclopedia of Genes and Genomes (KEGG, http://www.genome.jp/kegg), a major public pathway-related database [28, 29] that can analysis a gene product during metabolic processes and related gene functions in cellular processes, was used for pathway assignments in which BLASTX with an E-value threshold of 1.00E-5 was applied.

### Discovery the putative terpene synthase genes in *P*. *orientalis* and evolutionary analysis

Terpenes play an important role in the physiology of gymnosperms [30]. Unigenes with conserved region of all terpene synthase (TPS) subfamilies were identified in *P*. *orientalis* transcriptome and *Pinus taeda* genome by querying the PLAZA 2.5 green plants clade database where gene families were defined by comparative genomic analysis of 25 plant organisms covering a broad taxonomic range. And, TPS family members were also retrieved for *Selaginella moellendorffii*, a lycophyte with whole genome sequence available, from the PLAZA 2.5 database. From *P*. *orientalis* transcriptome, we detected 80 unigenes that showed significant similarities to known TPS genes; however, 37 were excluded due to redundancy (see S2 File). For *Pinus taeda*, 93 loci that showed significant similarities to known TPS genes (S2 File). Additionally, 33 TPS loci from *S*. *moellendourffii* were selected for subsequent evolutionary analyses (S2 File). Finally, a total of 306 TPSs from *P*. *orientalis*, *Pinus taeda*, *S*. *moellendourffii*, *Abies grandis* [30], *Picea abies* [30], *Pieca sitchensis* [30], *Pinus taeda* [30], *Ginkgo biloba* [30], *Taxus brevifolia* [30], and *Eucalyptus grandis* [31] were retrieved and analyzed. Amino acid alignments were made for all TPSs, using Clustalx version 2.0 [32] following standard parameters. The alignments were manually adjusted with a focus on diagnostic conserved regions, and the alignment was truncated to ensure sites homology. To create a phylogeny, we first determined which amino acid substitution model provided the maximum likelihood tree with the best AIC value (Akaike’s information criterion value, corrected for samples size), and using Phyml [33] to further test whether gamma distribution estimation and/or proportion of invariable sites estimation improved the AIC value. The JTT model with estimation of invariable sites and estimation of gamma distribution were used in the tree with the highest AIC value. The phylogeny of these multiple species TPS gene family was determined using 100 bootstrap replicates, and the phylogenies were visualized using FigTree v1.3.1 [34].

### SSR loci detection and marker design

The SSR Identification Tool (SSRIT; http://www.gramene.org/db/markers/ssrtool) [35] was used to detect SSRs >1 kb among the *P*. *orientalis* unigenes. The parameters were designed to identify perfect di-, tri-, tetra-, penta-, and hexa-nucleotide motifs, with minimum thresholds of 6, 5, 4, 4 and 4 repeats, respectively. QDD version 3.1 [36] and design primers for each uniquely occurring locus.

We provided parts of our data analysis pipeline in S3 File.

## Results

### Sequence data and assembly

In total, 110,826,650 raw reads were obtained, with an average length of 101 bp, totaling ~11.2 Gbp of sequence data (NCBI SRA accession No.: SRR1997784). We initially evaluated the base quality of the raw sequenced reads (S1 Fig) and the cleaned sequenced bases and removed poor quality reads (S2 Fig). The quality of reads per base, distribution of mean quality scores and the GC content distribution over all sequenced reads were compared against the theoretical GC distribution for our clean reads and were all at an acceptable level. After removal of the raw reads with adaptor fragments, ambiguous, and low-quality bases, 104,073,506 clean reads (average length 99.04 bp) totaling ~10.3 Gbp of sequence data remained and the quality of reads was sufficient to proceed to *de novo* assembly. The reads of *P*. *orientalis* were aligned against the draft genome sequences of *Picea abies*, *Picea glauca*, and *Pinus taeda* (S4 File). The results indicated that 72,562,967 (69.72%) reads can be mapped to the draft genome sequence of *Picea abies* and 68,839,841 (66.15%) mapped reads are properly paired. Similar proportions of mapped reads were observed in the other two alignments, 74,794,265 (71.87%) and 70,684,208 (67.92%) reads of *P*. *orientalis* could be mapped to the draft genome sequences of *Picea glauca* and *Pinus taeda*, respectively (S4 File). Comparison to the three conifer transcriptome sequences developed from whole genome annotation showed that most *P*. *orientalis* transcriptome reads can be mapped, and the proportion of mapped reads is similar. We observed 40,080,051 (38.51%), 47,755,138 (45.89%), 3,103,563 (2.98%), 4,013,571 (3.86%), and 9,860,656 (9.47%) *P*. *orientalis* reads were mapped to *Picea abies*, *Picea glauca*, *Pinus taeda* HQ.A (high quality, full length genes), *Pinus taeda* HQ.B (high quality, partial genes), and *Pinus taeda* LQ (low quality) transcriptomes, respectively (S5 File). These two sets of mapping experiments suggest that there are considerable genetic divergence between Cupressaceae and Pinaceae families.

**Fig 1:**
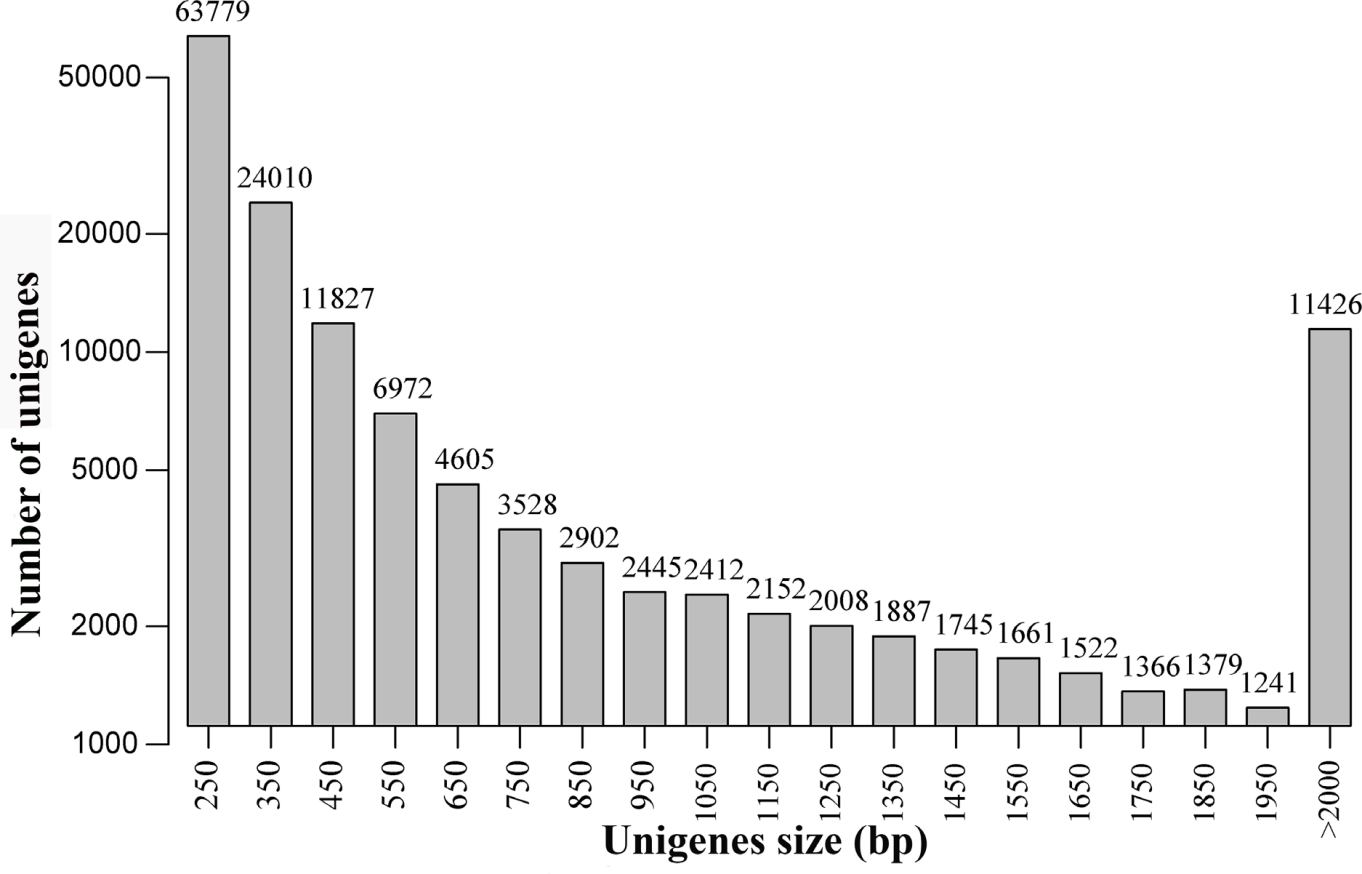
Length distributions of all unigenes.

The clean data was assembled *de novo* using Trinity and produced 228,948 transcripts ≥200 nt, with an average length of 946 nt. From the primary assembled transcripts, 148,867 unigenes ≥200 nt (S6 File) were obtained with an average length of 686 nt, with an N50 of 1,320 nt and an N90 of 259 nt. Of the unigenes, 49,330 were ≥500 nt and 28,822 were ≥1,000 nt (Table 1). Length distributions of all the unigenes were shown in Fig 1.

**Table 1:** *P*. *orientalis* assembled transcripts and unigenes properties summary.

	Transcripts	Unigenes
No. of reads > = 200 nt	228,948	148,867
No. of reads > = 500 nt	111,828	49,330
No. of reads > = 1000 nt	73,298	28,822
N50 (nt)	1,755	1,320
N90 (nt)	388	259
Total length (nt)	216,674,972	102,175,229
Max length (nt)	27,201	27,201
Min length (nt)	201	201
Average length (nt)	946.39	686.35
Sequencing depth (mean ± SD)		101±32735
Median sequencing depth		35

### Identification of core eukaryotic proteins

Several conserved core eukaryotic genes (CEGs), representing an unbiased set of proteins that are conserved in diverse eukaryotes, were identified in our transcriptome assembly. Using CEGMA, 811 full-length homologs of 245 (98.79%) out of 248 CEGs and 936 partial-length homologs of 247 (99.60%) out of 248 CEGs were identified in our assembly (S7 File).

### Assessment of gene completeness, homology to other conifer genomes and potential contamination

TRAPID predicted 35,480 (23.8%) full- or quasi full-length and 14,910 (10.0%) partial coding unigenes in the present assembly with assignment ratio of 2.38. However, there was 98,477 (66%) unigenes that did not significantly match any protein sequences in the PLAZA 2.5 green plants clade database. These un-annotated unigenes include undiscovered conifer genes, non-plant genes, as well as artefactual assemblies. To distinguish those possibilities, unigenes were blasted against the Nr database and mapped to the draft genome of *Pinus taeda* [18]. We found 4,334 (4.43%) of the un-annotated unigenes significantly matched plant genes, and 13,079 (13.37%) significantly matched non-plant genes (archaea 1; viruses 104 (0.11%), bactecia 10,232 (10.46%), and animal 2,743 (2.80%) (S8 File). Thus, we consider non-plant matched unigenes are representative of mostly contaminants from other organisms. Additionally, results of the un-annotated unigenes mapped to the *Pinus taeda* draft genome indicated that 2,297 (2.35%) shared significant homology with the pine genome (S8 File). Interestingly, 339 (0.23%) of the total unigenes produced significant hits with both of non-plant taxa in Nr and the pine genome.

When all *P*. *orientalis* unigenes were mapped to the *Pinus taeda* draft genome, 18.74% of mapping rate was found, and 2,561 unigenes can be aligned to two different contigs (S8 File), confirming that part of assembled unigenes were conifer transcripts and a distinct genetic divergence between Cupressaceae and Pinaceae species exists. This low mapping rate to the pine genome could possibly be attributed to the low quality of pine draft genome assembly which contains more 10 million contigs. Among the unigenes that produced significant matches in PLAZA 2.5 database, 25,451 (17.10% of total unigenes) had no match to *Pinus taeda* draft genome, signifying that some of these unigenes may be *P*. *orientalis* specific. We found 90,194 (60.59%) unigenes had at least one significant hit in any one of the three examinations with PLAZA, Nr, and pine genome and only 23,161 (15.56%) unigenes had significant hits in all examinations, indicating the existence of a large gap in conifers accumulated functional information.

### Functional annotation

Functional annotation of the unigenes against protein databases revealed a total of 62,938 (42.28%) unigenes annotated against Nr and 58,566 (39.34%) showed significant similarity to known proteins in the Pfam databases. Additionally, 42,158 (28.32%), 23,179 (15.57%), and 25,625 (17.21%) were annotated against the GO, KOG, and KEGG databases, respectively (Table 2).

**Table 2:** Unigene homology searches against the protein databases.

Database	Unigenes	Percentage
Nr	62,938	42.28%
Pfam	58,566	39.34%
KOG	23,179	15.57%
GO	42,158	28.32%
KEGG	25,625	17.21%

**Fig 2:**
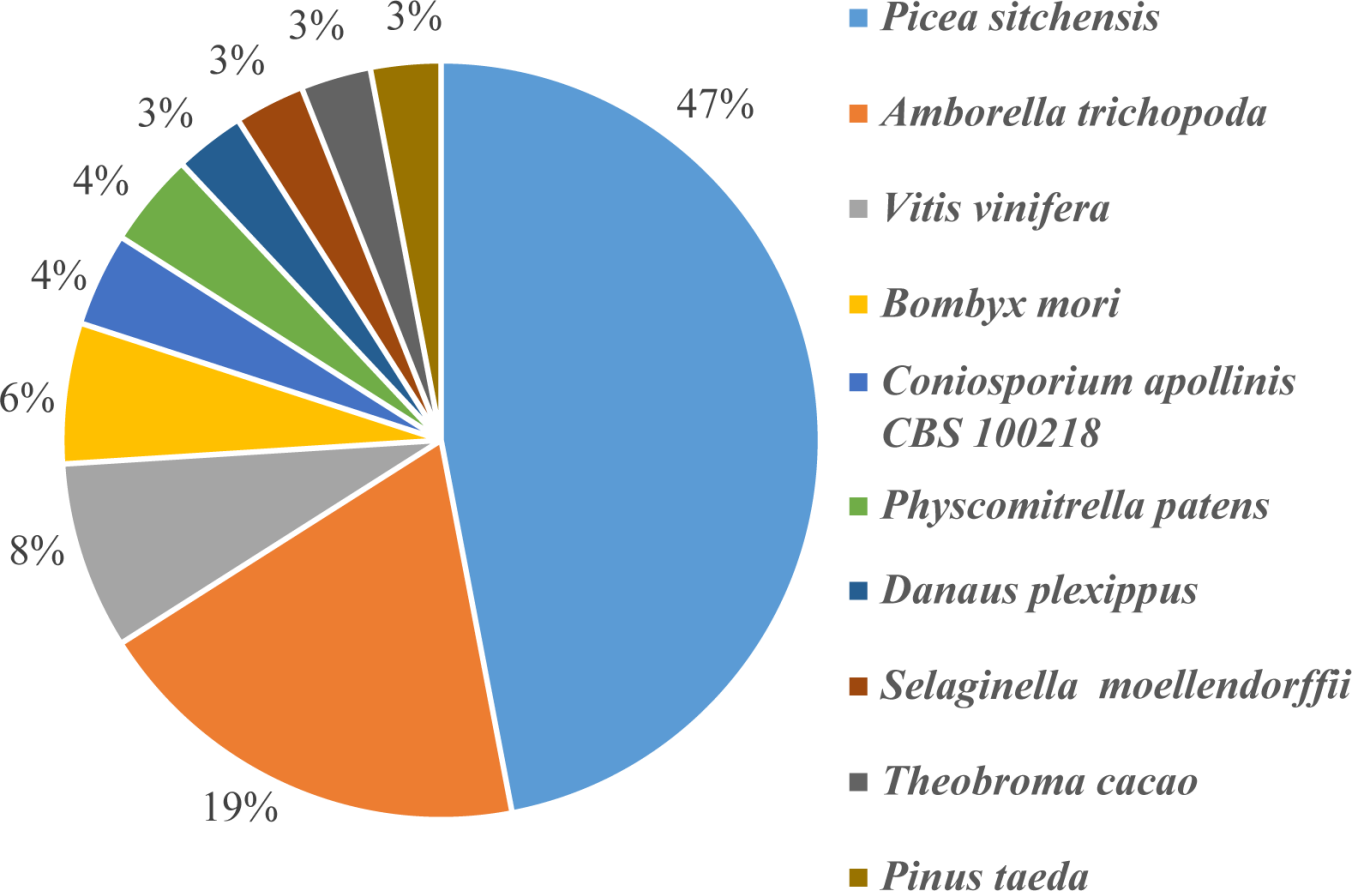
Distribution of the top BLASTX hits for unigenes in the Nr database.

The top-scoring BLASTX hits against the Nr protein database showed that the top three species were *Picea sitchensis* (47%), *Amborella trichopoda* (19%) and *Vitis vinifera* (8%), and accounting for 74% of the identified unigenes. The remaining 26% were distributed among seven species: *Bombyx mori* (6%), *Coniosporium apollinis CBS100218* (4%), *Physcomitrella patens* (4%), *Danaus plexippus* (3%), *Selaginella moellendorffii* (3%), *Theobroma cacao* (3%), and *Pinus taeda* (3%) (Fig 2).

**Fig 3:**
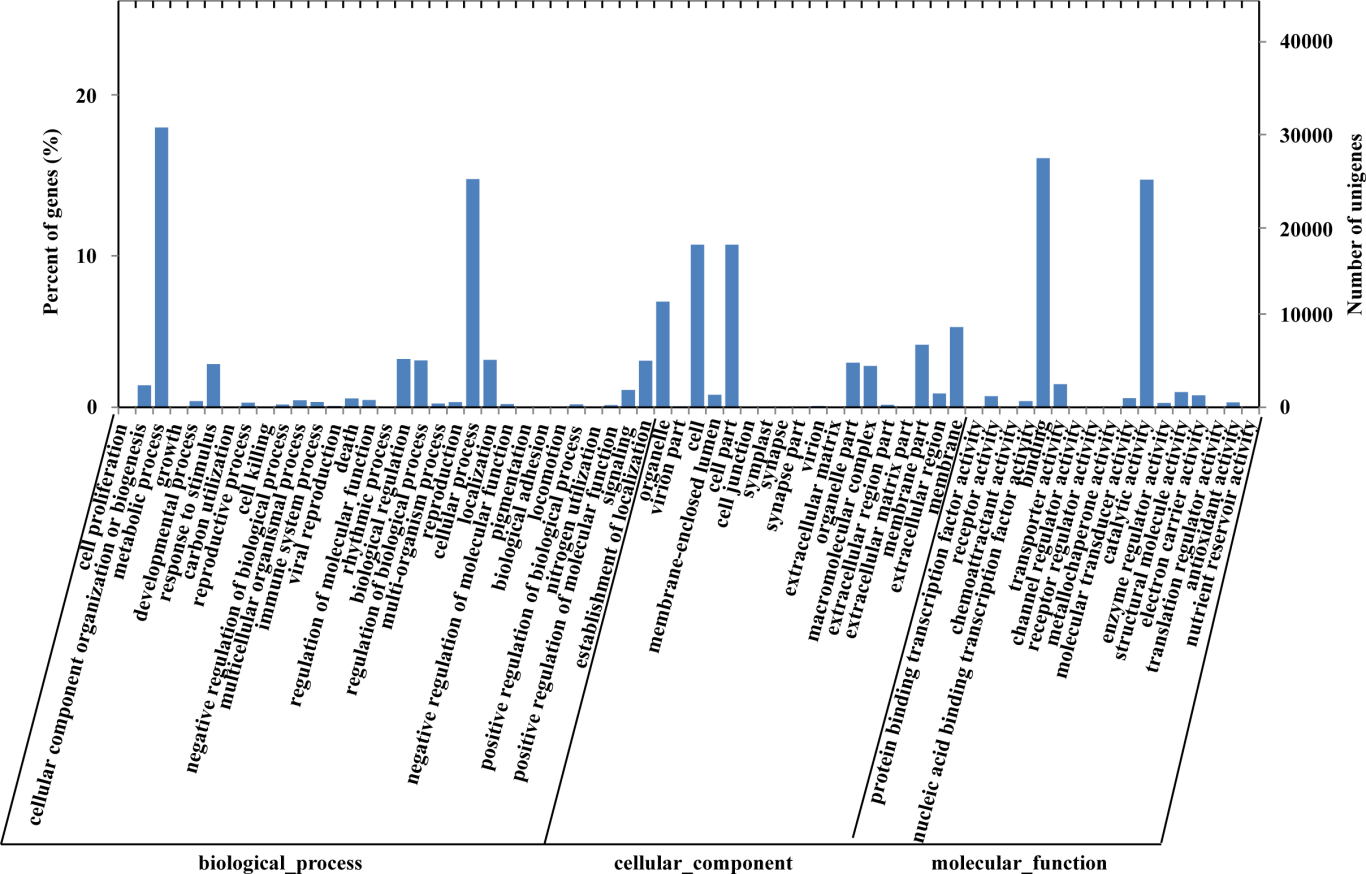
GO classification of *P*. *orientalis* unigenes.

GO terms were assigned to unigenes for functional categorization (S9 File). In total, 42,158 (28.32%) unigenes were categorized into 66 subcategories and are grouped in three main groups; namely, 1) biological process (31 subcategories), 2) cellular component (18), and 3) molecular function (17). Some of these unigenes were annotated with multiple GO terms; biological process was the largest cluster comprised of 92,660 annotations, followed by cellular component (76,051), and molecular function (62,063) (Fig 3). Within the biological process category, metabolic process (30,758) and cellular process (25,083) were prominent, indicating that these unigenes are involved in important metabolic activities. In the cellular component category, 17,871 and 17,861 unigenes were assigned to the cell and cell part, respectively, and represented the majority of the unigenes in this category. However, only 29 unigenes were assigned to the symplast (8), synapse (13) or synapse part (8). In the molecular function category, the dominant GO terms were grouped into binding (27,379) and catalytic activity (25,015), followed by transporter activity (2,547), structural molecule activity (1,660) and electron carrier activity (1,325). These GO annotations showed that diverse structural, regulatory, metabolic, and transporter proteins are encoded by genes expressed in *P*. *orientalis*.

**Fig 4:**
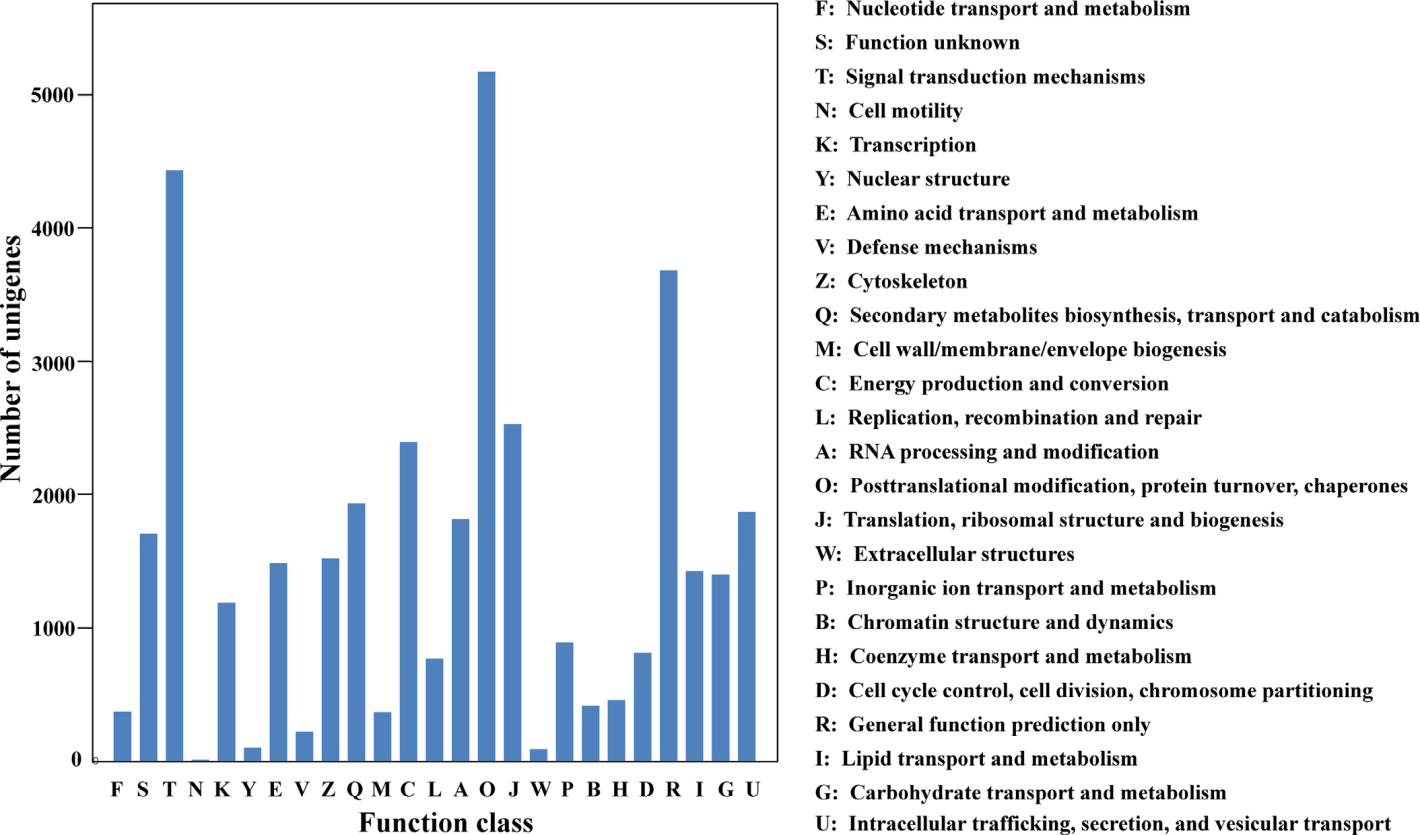
KOG classification of *P*. *orientalis* unigenes.

To further predict the function of the genes and to evaluate the classification of their origins, all the assembled unigenes were searched against the KOG database to classify their products in clusters of orthologs or paralogs. Overall, 23,179 (15.57%) unigenes were assigned to 25 KOG functional categories (Fig 4). The post-translational modification, protein turnover, and chaperones category represented the largest group (5,178; 3.48% of all unigenes), followed by signal transduction mechanisms (4,436; 2.98%), and general function prediction only (3,684; 2.47%). Fewer unigenes were assigned to extracellular structures and cell motility (89 and 9, respectively).

**Fig 5:**
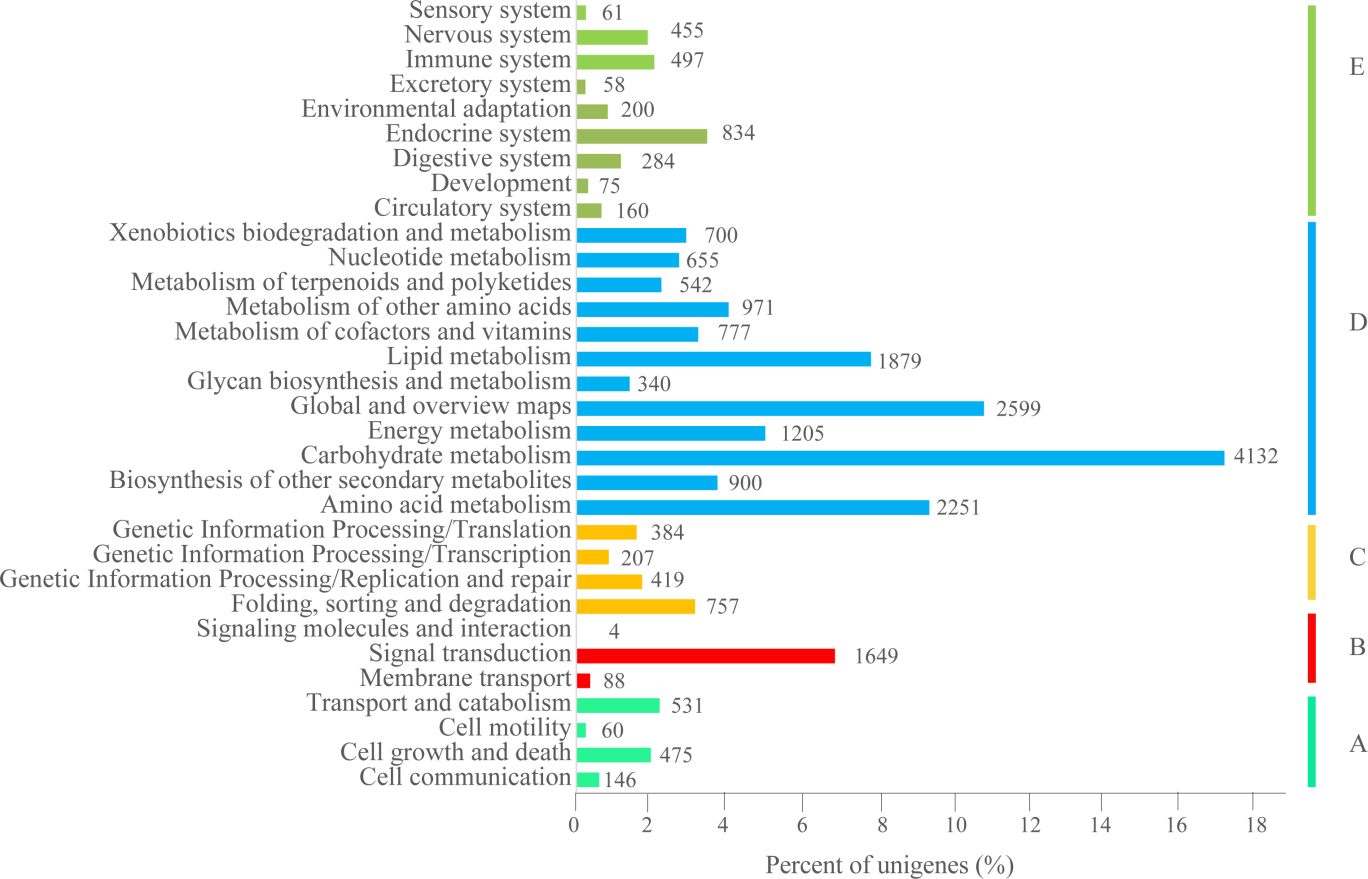
KEGG classification of the assembled unigenes. A total of 25,625 unigenes matched with BLASTX hits and 24,295 were assigned to five KEGG biochemical pathways: cellular processes (A), environmental information processing (B), genetic information processing (C), metabolism (D) and organismal system (E).

To evaluate the biological pathways that might be active in *P*. *orientalis*, the unigenes were aligned to those in the KEGG database [37] and of the 148,867 unigenes, 25,625 (17.21%) produced significant matches and were assigned to 322 KEGG pathways (S10 File). Of these, 24,295 were assigned to the following five KEGG biochemical pathways: 1) cellular processes (1,212 unigenes, Fig 5A), 2) environmental information processing (1,741, Fig 5B), 3) genetic information processing (1,767, Fig 5C), 4) metabolism (16,951, Fig 5D), and 5) organismal systems (2,624, Fig 5E). Pathways 1 to 4 were classified as carbohydrate metabolism (4,132 unigenes), global and overview maps (2,599), amino acid metabolism (2,251), and lipid metabolism (1,879). Energy metabolism (1,205) followed by signal transduction (1,649) comprised the largest metabolic groups involved in environmental information processing (Fig 5). These functional annotations will provide a valuable resource for further exploration of specific biological processes, functions, structures, and pathways of gene products in *P*. *orientalis*.

### Discovery of putative TPS genes from the *P*. *orientalis*

**Fig 6:**
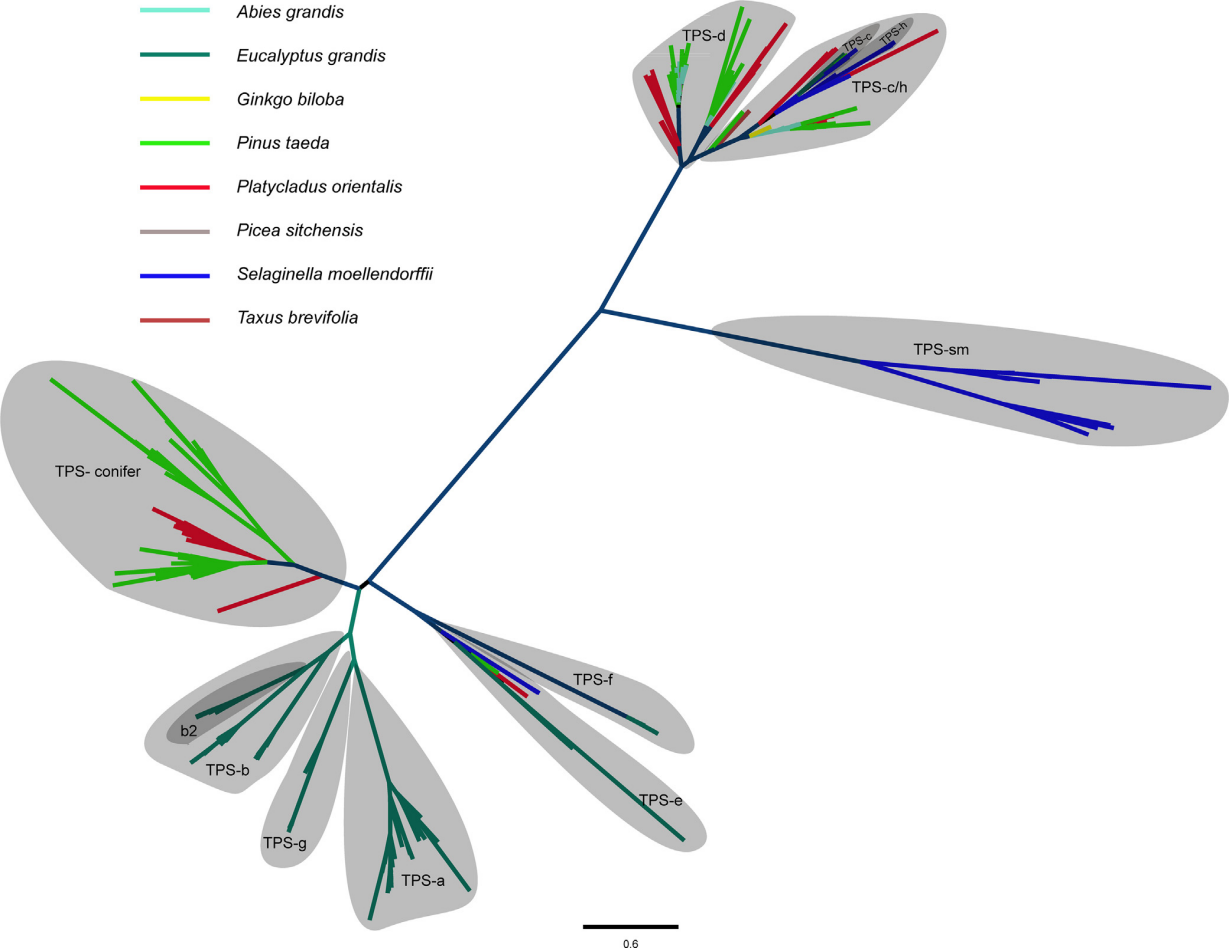
Phylogenetic tree of the putative TPSs from *P*. *orientalis* transcriptome and representative characterized TPSs from a broad of plant lineages. Nine subfamilies (groups) of TPSs were reconstructed, with two subfamilies (TPS-conifer and TPS-sm) were firstly recognized.

The putative terpene synthase (TPS) protein sequences were divided into seven subfamilies by previous evolutionary analyses, designated TPS-a through TPS-h [30–31, 38–40]. The present study, have substantially extended these analyses to include 43 new TPS unigenes from *P*. *orientalis* and 93 genes from *Pinus taeda* (Fig 6, S2 and S11 Files). Nine subfamilies of TPSs were reconstructed, with two firstly recognized subfamilies. These include three angiosperm-specific subfamilies TPS-a, TPS-b and TPS-g. Subfamily TPS-h (formerly defined as *S*. *moellendorffii* specific) and subfamily TPS-c (most conserved among land plants) connected closely to each other and formed one larger group with TPSs from conifer species, here we named this emerging group TPS-c/h subfamily. Forty TPSs from *P*. *orientalis* and *Pinus taeda* are assigned to subfamily TPS-d (previously identified as gymnosperm specific). The two subfamilies TPS-e and TPS-f which are conserved among vascular plants are grouped with TPSs from all vascular taxa. A new subfamily (TPS-conifer, the present study) which is distinct from other subfamilies was reconstructed, and this subfamily is formed by TPSs from only 16 unigenes of *P*. *orientalis* and 51 genes of *Pinus taeda*. The other new subfamily was named as TPS-sm which consists of 19 TPSs from *S*. *moellendorffii*.

### Environmental signaling and stress response genes in *P*. *orientalis*

In this study, 1,941 unigenes were identified to be involved in environmental signaling and stress responses. Signal transduction pathways, such as ABC transporters, cAMP signaling, and AMPK signaling, etc., play important roles in stress response. Additionally, 200 unigenes were annotated to five main environmental adaptation pathways. In these pathways, plant-pathogen interaction contained the largest number of unigenes (131 unigenes). Circadian rhythm plant formed the second largest environmental adaptation pathway (34). The third largest environmental adaptation pathway was circadian rhythm-fly (14), followed by other environmental adaptation pathways, circadian entrainment (12), and circadian rhythm (9). In these pathways, the circadian system is an endogenous rhythm over an approximate 24h period that provides temporal organization of biological activities. This pathway is also important in the control of plant physiology and forms a vital part of the plant resistance pathway. We speculate that this pathway is related to the capability of *P*. *orientalis* to withstand harsh environments and related to pathogens resistance.

**Fig 7:**
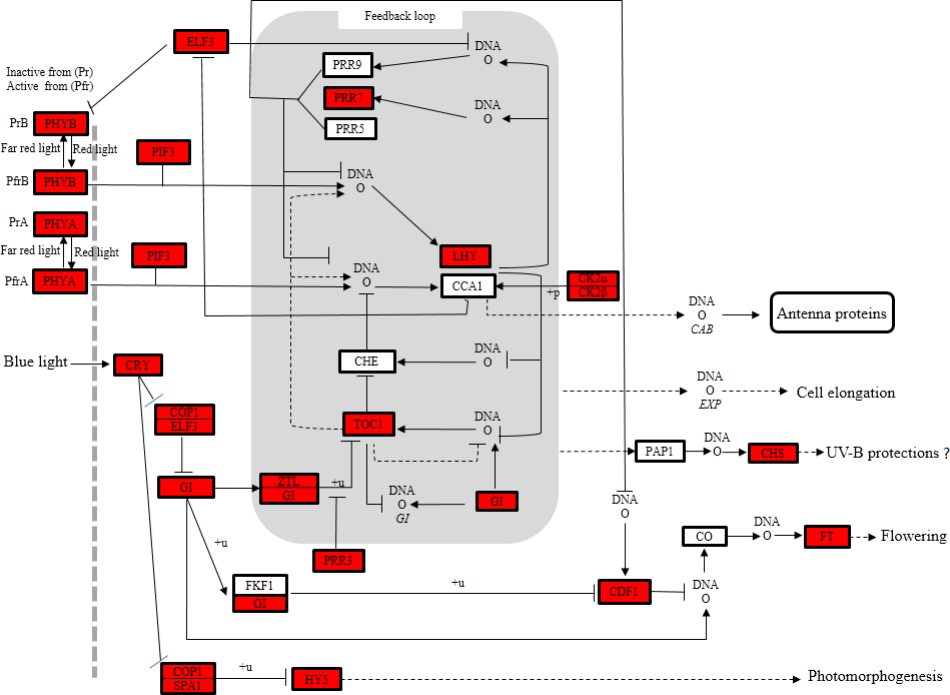
Metabolic pathway of the circadian rhythm for the unigenes identified in *P*. *orientalis*. Each box represents the substance involved in each section of the pathway. The red boxes represent substances assigned at least one unigene.

A circadian rhythm pathway was identified in the KEGG pathways involving 34 unigenes, which were related to 19 substances and were identified in the present annotated *P*. *orientalis* transcriptome database. The number of unigenes annotated for these 19 substances varied from 2 to 16, and two important enzymes (COP1 [EC: 6.3.2.19] and CHS [EC: 2.3.1.74]) involved in the circadian rhythm pathway were also annotated. The metabolic pathway and annotated unigenes for the circadian rhythm are shown in Fig 7. Each element in the pathway is associated with several unigenes and this resolved pathway will be useful in further studies of environmental signaling and stress response mechanisms in *P*. *orientalis*.

### Characterization of SSRs and marker development

The 28,822 unigene sequences >1 kb were used for SSR identification based on 148,867 examined sequences. In total, 4,715 of these sequences contained 5,296 SSRs of 142 motif types. The frequency of SSRs in the *P*. *orientalis* transcriptome was 1 per 1.3 kb (Table 3). Di-nucleotide repeats were most abundant, accounting for 70.31% (1,279), followed by tri- (522, 28.70%) and tetra-nucleotide (16, 0.88%) repeat motifs. Frequencies of SSRs with different numbers of tandem repeats were also calculated. SSRs with 5 tandem repeats (1,188, 65.31%) were most common, followed by 6 (293, 16.11%), 7 (145, 7.97%), 8 (57, 3.13%), and 9 tandem repeats (53, 2.91%). A detailed list of SSRs identified is shown in Table 4. Using QDD, 1,376 unique SSR-containing unigenes were identified and 14,996 pairs of primers for 893 SSR loci were designed. Unigenes containing SSRs and primers targeting the SSRs are listed in S12 File.

**Table 3:** *P*. *orientalis* transcriptome generated simple sequence repeats (SSR).

No. of unigenes longer than 1 kb	28,822
Total nucleotides screened (kb)	26,360
No. of unigenes containing SSRs	4,715
No. of identified SSRs loci	5,296
SSR motif types	142
Frequency of SSR in transcriptome	1/1.3kb

**Table 4:** Frequency of simple sequence repeats (SSR) in the transcriptome of *P*. *orientalis*.

Motif length	Repeat numbers									Total	%
	5	6	7	8	9	10	11	12	>12		
Di	836	171	86	50	53	40	40	3		1279	70.31
Tri	340	116	59	7						522	28.70
Tetra	10	6								16	0.88
Penta	2									2	0.11
Hexa											
**Total**	1188	293	145	57	53	40	40	3		1819	
**%**	65.31	16.11	7.97	3.13	2.91	2.20	2.20	0.16			

## Discussion

High-throughput RNA sequencing is a useful approach to obtaining a complete set of transcripts from selected tissues for species of interest at specific developmental stages, or under varying physiological conditions [12]. Because of the potential advantages of high throughput technologies, i.e., accuracy and low cost, numerous transcriptomes from non-model species have recently been sequenced by NGS technologies in combination with multiple bioinformatics approaches [41, 42]. This approach was used in the present study to construct the transcriptome of *P*. *orientalis*, a widespread coniferous tree used in ecological restoration and of economic value and for which very little molecular information is available. The aim of this study was to provide comprehensive transcriptome data to facilitate genomic studies of *P*. *orientalis*. We aimed to sample all the major tissues using RNA-Seq to obtain a set of representative global transcripts of *P*. *orientalis*. Combined with extensive homology analyses, this resulted in a general understanding of the gene distributions in different pathways and enabled identification of a large set of SSR markers for practical applications in breeding programs and provenance and pedigree tracking. We believe the availability of these transcriptome data for *P*. *orientalis* will meet the informational needs for molecular genetic studies of this species and its relatives.

In the present study, ~10.41 million high quality reads were assembled into 148,867 unigenes, with an average sequence length of 686 bp. Quality assessment using TRAPID and CEGMA showed that the transcriptomes obtained for *P*. *orientalis* were mostly complete for highly conserved core eukaryotic genes. The *P*. *orientalis* transcriptome was compared with other conifer transcriptomes released recently. The properties of these transcriptomes are summarized in S13 File. The *de novo* assembly of *P*. *orientalis* transcriptomes had high coverage of unigenes and assembled sequence lengths. The total length of all unigenes was 102,175,229 nt and the number of unigenes was also the largest (148,867) among studied conifers. The longest assembled sequence (27,201 nt) was again found in *P*. *orientalis*. Regarding assembly quality, the longer the N50 and the shorter the N90, the better the quality of the transcriptome data. The N50 of the present transcriptome assembly was 1,320, which is the second largest and the N90 was 259, which is the second smallest as compared to published information from other conifers (S13 File). Altogether, the comparison result of transcriptome’s generic parameters implied that the transcriptome of *P*. *orientalis* is at a high quality level.

The complete set of conifer transcriptomes was re-analyzed by TRAPID (http://bioinformatics.psb.ugent.be/webtools/trapid/trapid) which is an efficient online tool for the functional and comparative analysis of RNA-Seq transcriptomes (see S13 File). The result of this re-analysis showed that the percentage of meta annotation “full-length” and meta annotation “quasi full-length” of *P*. *orientalis* unigenes were 13.6% (20,249 unigenes) and 10.2% (15,231), which all at a high quality level among available transcriptomes data (S13 File). Additionally, other general statistic parameters of *P*. *orientalis* unigenes were 14,910 (10%), 98,477 (66.2%), 5,514, 42,700 (28.7%), 5,458, and 46,726 (31.4%) for meta annotation “partial”, meta annotation “no information”, GO terms, transcripts with GO, interPro domains, and transcripts with protein domain, respectively. This comparison illustrated that the parameters of the *P*. *orientalis* transcriptome data are at a reasonable level or even better than previously published conifers transcriptomes. Furthermore, the present results not only validate the high quality and reliability of the *P*. *orientalis* transcriptome it also confirmed the presence of a significant homology to other conifers, thus we expect it will provide good basis for qualitative genomics traits analyses and the development of marker-aided breeding programs [43–46] for the species. Nevertheless, *de novo* assembly also has its own problems, e.g. contaminants from other organisms, false positives assembled transcripts. The assembled transcriptome datasets lacked the ability to distinguish and classify the lower confidence annotations, which is beyond the scope of this study, and this can be resolved once a genome-based prediction of gene models is available. It is noteworthy to mention that 8.80% of total unigenes significantly matched non-plant genes in Nr database, which indicates potential contamination. Furthermore, the low mapping rate of both short reads (67.92%) or unigenes (18.74%) from *P*. *orientalis* transcriptome to *P*inus *taeda* genome signifies considerable genetic divergence between Cupressaceae and Pinaceae species.

Terpenes, as important secondary compounds in plants, play a significant role in species interaction with its biotic and even abiotic environment [30]. Generally, plants that generate or store few terpenes, have only a small number of TPS genes, such as *Arabidopsis thaliana* (32 putative functional and 8 pseudo TPS genes) [47] and *Poplar* (38 putative functional genes) [48]. We identified 43 unigenes in the *P*. *orientalis* transcriptome and 93 genes in *Pinus taeda* all of which have a high sequence similarity to known terpene synthase genes from other species, suggesting that terpenes may play an important role in physiology of *P*. *orientalis* and conifer. Besides, we reconstructed two unidentified but significant TPS subfamilies and extended new TPS sequences to subfamily TPS-h which may include other gymnosperm TPS sequences.

*P*. *orientalis* is highly adaptable to harsh environmental conditions [1] and a total of 200 unigenes were annotated to five main environmental adaptation pathways. These findings are important for the study of the interaction mechanism between *P*. *orientalis* and its environment. We are especially interested in these groups of unigenes for future population genetic studies to explore the adaptability of *P*. *orientalis* over a range of diverse environments. Additionally, we identified numerous essential structural genes involved in the plant resistance pathway and circadian rhythm. These findings are important in enabling the optimum conservation and utilization of this resource and their inclusion in future breeding programs. It should be pointed out that while the present study succeeded in providing the functional annotation of the discovered unigenes (i.e., predicted function), further studies are required for their functional validation.

SSRs are highly efficient genetic markers and are extensively used in molecular breeding research and genetic mapping [49–53]. The traditional methods for developing SSR markers can be time-consuming and the present study clearly illustrated the utility of NGS methods in producing large amounts of sequence data from large-scale transcriptome sequencing, enabling the efficient, convenient, and low-cost development of SSR markers from transcriptome data. Therefore, the development of a large set of EST-SSRs is essential for fingerprinting and parentage analyses of *P*. *orientalis* and closely related species. The transcriptome database of *P*. *orientalis* contains a large set of functionally-related EST-SSR markers, which will provide useful tools for assessing genetic variation and relationships in genetic mapping studies [54].

## Conclusion

This study provides a global set of transcripts for *P*. *orientalis* and represents the first *de novo* assembled transcriptome for this species. In total, 148,867 unigenes with high sequence qualities were obtained. These unigenes were used in BLASTX searches and for annotation against public databases, and were then functionally classified based on BLASTX searches. These results provide comprehensive coverage to enable the discovery of genes known to be involved in environmental signaling and stress responses. This unigenes dataset will speed up genomic research of the species and aid in understanding of the environmental adaptation mechanisms of *P*. *orientalis* and its regulation of the production of chemical compounds. We believe that this transcriptome dataset will serve as an important public information platform to improve our understanding of the molecular genetics of *P*. *orientalis* and other closely related species.

## Supporting Information

S1 Fig Assessment of reads by FastQC before quality control. a) Quality of raw-reads per base. The central red line is the median base quality (the yellow box represents the interquartile range (25–75%), the upper and lower whiskers represent the 10 and 90% points, respectively, and the blue line represents the mean base quality), b) Distribution of the mean quality scores over all sequenced reads, and c) The distribution of GC content over all sequenced reads compared against the theoretical GC distribution (the blip in the GC content above the theoretical GC distribution is most likely due to the primers utilized at the 5' end of the reads during RNA-Seq library preparation and sequencing).
(TIF)

S2 Fig Assessment of reads using FastQC after quality control. a) Quality of reads per base after adaptive window trimming using a quality average threshold of 20 and a minimum length threshold of 20 (the central red line is the median value, the yellow box represents the interquartile range (25–75%), the upper and lower whiskers represent the 10 and 90% points, respectively, and the blue line represents the mean base quality), b) The mean sequence quality scores over all reads, and c) The GC content distribution over all sequenced reads compared against the theoretical GC distribution.
(TIF)

S1 File Examples of how “partial”, “full” and “quasi-full” length transcript were defined. (DOCX)

S2 File The terpene synthase (TPS) proteins analyzed in phylogenetic analysis. (DOCX)

S3 File The parts of our data analysis pipeline. (TXT)

S4 File The results of the *P*. *orientalis* reads aligned against the draft genome sequences of *Picea abies*, *Picea glauca*, and *Pinus taeda*. (XLSX)

S5 File The results of the *P*. *orientalis* reads aligned against the transcriptomes of *Picea abies*, *Picea glauca*, *Pinus taeda*. (XLSX)

S6 File All the unigenes of *P*. *orientalis* in this study. (ZIP)

S7 File Results of the gene completeness assessment of our transcriptome assembly from CEGMA. (TXT)

S8 File Results showing all *P*. *orientalis* unigenes mapped to the *Pinus taeda* draft genome, PLAZA 2.5 and Nr database. (XLSX)

S9 File The results of GO terms annotation. (XLSX)

S10 File All unigenes pathway annotation. (XLSX)

S11 File A different representation of the phylogenetic tree of terpene synthase (TPS) gene family with bootstrap values. (PDF)

S12 File Results showing the original unigenes, the target SSR region and the primer properties. (TXT)

S13 File The characteristics of the transcriptome assemblies of related conifer species and the re-analysis results from TRAPID. (XLSX)
