# Supplementary figures and images for: De Novo Transcriptome Assembly and Characterization for the Widespread and Stress-Tolerant Conifer Platycladus orientalis
Source: PLoS One. 2016 Feb 16;11(2):e0148985. doi: 10.1371/journal.pone.0148985 (PMC4755536; doi:10.1371/journal.pone.0148985)

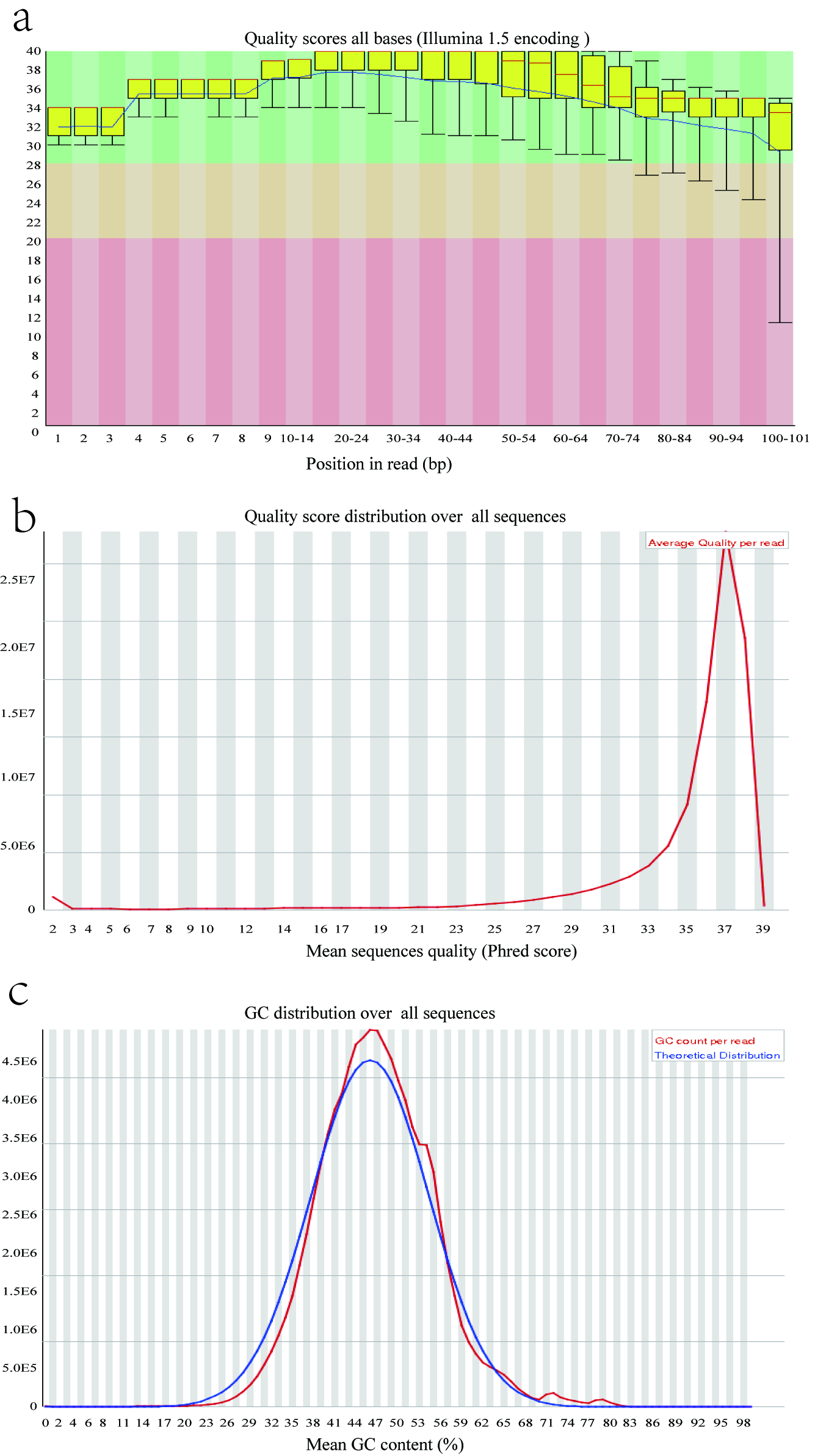

Supplement: S1 Fig — a) Quality of raw-reads per base. The central red line is the median base quality (the yellow box represents the interquartile range (25–75%), the upper and lower whiskers represent the 10 and 90% points, respectively, and the blue line represents the mean base quality), b) Distribution of the mean quality scores over all sequenced reads, and c) The distribution of GC content over all sequenced reads compared against the theoretical GC distribution (the blip in the GC content above the theoretical GC distribution is most likely due to the primers utilized at the 5' end of the reads during RNA-Seq library preparation and sequencing). (TIF) [file pone.0148985.s001.tif]

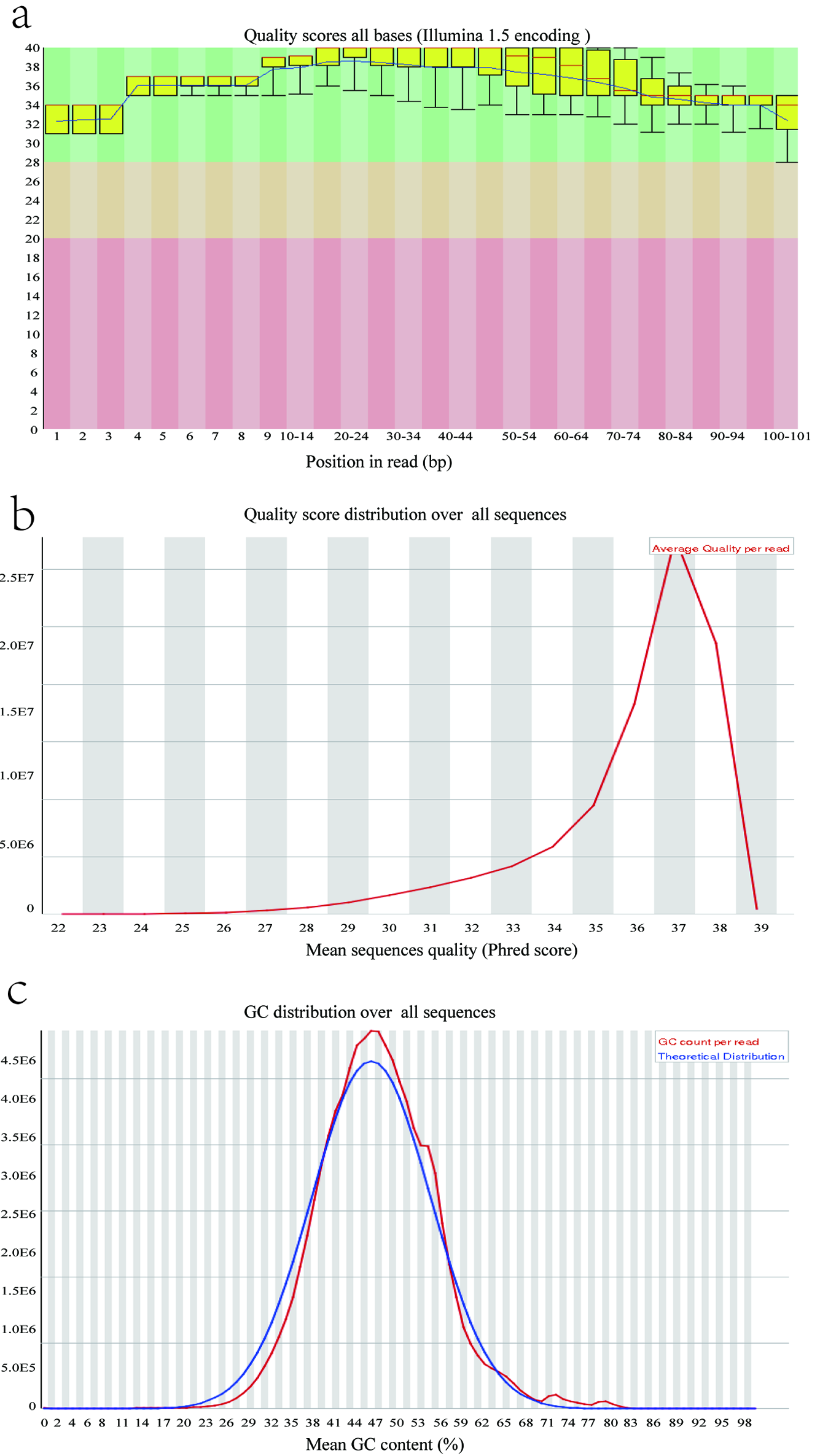

Supplement: S2 Fig — a) Quality of reads per base after adaptive window trimming using a quality average threshold of 20 and a minimum length threshold of 20 (the central red line is the median value, the yellow box represents the interquartile range (25–75%), the upper and lower whiskers represent the 10 and 90% points, respectively, and the blue line represents the mean base quality), b) The mean sequence quality scores over all reads, and c) The GC content distribution over all sequenced reads compared against the theoretical GC distribution. (TIF) [file pone.0148985.s002.tif]

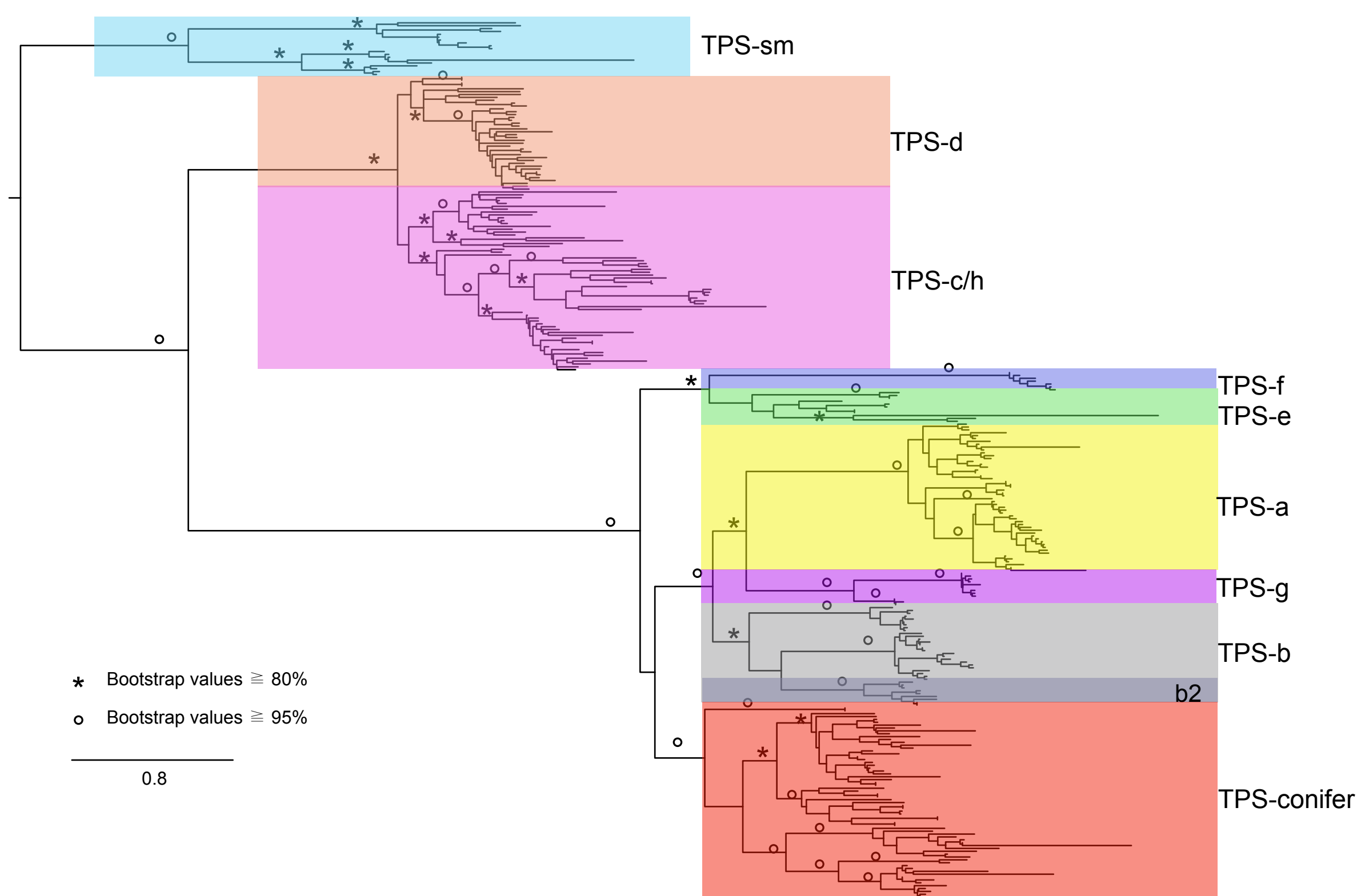

Supplement: S11 File — (PDF) [file pone.0148985.s013.pdf]
