# Supplementary material for: De Novo Transcriptome Assembly and Characterization for the Widespread and Stress-Tolerant Conifer Platycladus orientalis
Source: PLoS One. 2016 Feb 16;11(2):e0148985. doi: 10.1371/journal.pone.0148985 (PMC4755536; doi:10.1371/journal.pone.0148985)
Supplement: S1 File — (DOCX) [file pone.0148985.s003.docx]

**Examples of how “partial”, “full” and “quasi-full” length transcript were defined**

In TRAPID (http://bioinformatics.psb.ugent.be/webtools/trapid/) processing of transcriptome assembly, large-scale sequence similarity searches and open-reading frame (ORF) detection are combined to identify coding sequences, assign transcripts to gene families, identify partial/full length transcripts, and generate homology-based functional annotations. Please refer to the original paper on TRAPID [1] for details of the whole process. Here, we just give simple examples of how “partial”, “full” and “quasi-full” length transcript were defined.

1. **“Partial” length transcript**

We have a transcript named “**comp100012_c0_seq1**” which has 208 nt in length. An ORF (**Open-reading frame**) of 207 nt (69 aa) was detected from this transcript, and the transcript was assigned to the gene family (HOM000023). TRAPID identifies this transcript with an ORF > 2 deviations shorter than the average ORF length of the assigned gene family (excluding the 10% longest and shortest sequences within the family), so the transcript (comp100012_c0_seq1) receives the label of “**Partial**”.

**The sequence of the transcript and the ORF detected**

**>comp100012_c0_seq1** Sequence length: 208 nt

TCCAATCATAGGAACTCCCAAACTAATGGCCTCCATGGTAGAGTTCCATCCATTATGTGTAAGAAAACCTCCTACAGATGGGTGGGAAAGAACCTCCATCTGCTTCGTCCAACTAACAATCAGGGCTCGATCCTTGGTTCGATCGATGAAGCCCTCTGGTAATTCCACTGGTTTCCCTTCTGCCATATCTGATCTCAGAACCCACAAG

**> comp100012_c0_seq1_Open-reading frame (ORF)** Sequence length: 207 nt

TTGTGGGTTCTGAGATCAGATATGGCAGAAGGGAAACCAGTGGAATTACCAGAGGGCTTCATCGATCGAACCAAGGATCGAGCCCTGATTGTTAGTTGGACGAAGCAGATGGAGGTTCTTTCCCACCCATCTGTAGGAGGTTTTCTTACACATAATGGATGGAACTCTACCATGGAGGCCATTAGTTTGGGAGTTCCTATGATTGGA

1. **“Quasi Full Length” transcript**

We have a transcript named “**comp100055_c0_seq1**” which has 203 nt in length. An ORF (**Open-reading frame**) of 201 nt (67 aa) was detected from this transcript, and the transcript was assigned to the gene family (HOM000053). TRAPID identifies the transcript with an ORF longer than the mean minus 2 deviations, besides, the ORF sequence does not start with a start codon and not ends with a stop codon, so the transcript (comp100055_c0_seq1) receives the label “**Quasi Full Length**”.

**The sequence of the transcript and the ORF detected**

**>comp100055_c0_seq1** Sequence length: 203 nt

TCCCAATAGTGAAACTCGATCCAAAGAAATTTTCGATCTAATACATTCCAATGTTCACTGGCTTATGTCAGTGGTATCAATGAATGGTGCATCCTATTATGTGACCTTCATTGATGATTTCTCCAGGAAGACTTGTATCTACTTCATGAAGACCAAAGATGAGGCCTTTAGTCTATTCAAGGAGTTCAAAGATCAAGTGGAGG

**> comp100055_c0_seq1_Open-reading frame (ORF) detection sequence** Sequence length: 201 nt

CCCAATAGTGAAACTCGATCCAAAGAAATTTTCGATCTAATACATTCCAATGTTCACTGGCTTATGTCAGTGGTATCAATGAATGGTGCATCCTATTATGTGACCTTCATTGATGATTTCTCCAGGAAGACTTGTATCTACTTCATGAAGACCAAAGATGAGGCCTTTAGTCTATTCAAGGAGTTCAAAGATCAAGTGGAG

1. **“Full Length” transcript**

We have a transcript named “**comp100433_c0_seq1**” which has 357 nt in length. An ORF (**Open-reading frame**) of 234 nt (78 aa) was detected from this transcript, and the transcript was assigned to the gene family (HOM000053). TRAPID identifies the transcript with an ORF longer than the mean minus 2 deviations, and the ORF sequence starts with a start codon and ends with a stop codon, so the transcript (comp100433_c0_seq1) receives the label “**Full Length**”.

**>comp100433_c0_seq1** Sequence length: 357 nt

CTAGTCATTGGTATCACCCTTCATCTGTTCCCTGAGAGTTTGCCCACCTTTGTGACCGAGTTCCTGATACCCTTCATGCCCCAACTGCTCCTTTCGACTCTGCCCTCCTTTACTCCTACCCTCTGCAAGGCGTTCCTGGGCTTCCAAGCTCTTTCCACCAGTACCACCTGGTACAACAGTCTCTCGGGCCCTCGCCTTTTCATCCAACTGCTTTCTATCCTGCTCAGACGCCATGCTTTCTTCCTTCCAAACTAATCTGCTGATCTGATTCTGGCCCTCATAGTTATTTGGGTATTTATATGTGTTGCAAAAGAGGGAAGATAAGGTTGGTGGGGGTACAGGTGTAAGCTACGTGGG

**> comp100433_c0_seq1­_Open-reading frame (ORF) detection sequence** Sequence length: 234 nt

ATGGCGTCTGAGCAGGATAGAAAGCAGTTGGATGAAAAGGCGAGGGCCCGAGAGACTGTTGTACCAGGTGGTACTGGTGGAAAGAGCTTGGAAGCCCAGGAACGCCTTGCAGAGGGTAGGAGTAAAGGAGGGCAGAGTCGAAAGGAGCAGTTGGGGCATGAAGGGTATCAGGAACTCGGTCACAAAGGTGGGCAAACTCTCAGGGAACAGATGAAGGGTGATACCAATGACTAG

References:

1. Van Bel M, Proost S, Van Neste C, Deforce D, Van de Peer Y, Vandepoele K. TRAPID: an efficient online tool for the functional and comparative analysis of *de novo* RNA-Seq transcriptomes. Genome Biology. 2013;14(12):R134.
