# Supplementary material for: De Novo Transcriptome Assembly and Characterization for the Widespread and Stress-Tolerant Conifer Platycladus orientalis
Source: PLoS One. 2016 Feb 16;11(2):e0148985. doi: 10.1371/journal.pone.0148985 (PMC4755536; doi:10.1371/journal.pone.0148985)
Supplement: S2 File — (DOCX) [file pone.0148985.s004.docx]

| **Speices** | **Protein ID/Accession** | **Note** |
| --- | --- | --- |
| *Platycladus orientalis*^1^ | comp70221_c0_seq2 |  |
|  | comp70780_c0_seq1 |  |
|  | comp54945_c0_seq2 |  |
|  | comp31991_c0_seq1 |  |
|  | comp48210_c0_seq1 |  |
|  | comp55188_c0_seq2 |  |
|  | comp65931_c0_seq25 |  |
|  | comp49874_c0_seq1 |  |
|  | comp50685_c0_seq2 |  |
|  | comp53471_c0_seq3 |  |
|  | comp59790_c0_seq5 |  |
|  | comp96973_c0_seq1 |  |
|  | comp32024_c0_seq1 |  |
|  | comp55667_c0_seq1 |  |
|  | comp48752_c0_seq1 |  |
|  | comp55908_c0_seq1 |  |
|  | comp26306_c0_seq1 |  |
|  | comp26608_c0_seq2 |  |
|  | comp74403_c0_seq32 |  |
|  | comp74423_c0_seq15 |  |
|  | comp67513_c0_seq2 |  |
|  | comp68003_c0_seq1 |  |
|  | comp145421_c0_seq1 |  |
|  | comp74047_c0_seq56 |  |
|  | comp70990_c0_seq1 |  |
|  | comp66658_c0_seq1 |  |
|  | comp42687_c0_seq1 |  |
|  | comp55599_c0_seq2 |  |
|  | comp65812_c3_seq7 |  |
|  | comp37368_c0_seq1 |  |
|  | comp65364_c0_seq2 |  |
|  | comp42121_c0_seq1 |  |
|  | comp61490_c0_seq5 |  |
|  | comp48565_c0_seq1 |  |
|  | comp54721_c0_seq1 |  |
|  | comp43377_c0_seq1 |  |
|  | comp52707_c0_seq4 |  |
|  | comp71289_c0_seq3 |  |
|  | comp74623_c0_seq7 |  |
|  | comp74603_c0_seq2 |  |
|  | comp6969_c0_seq1 |  |
|  | comp146301_c0_seq1 |  |
|  | comp73825_c0_seq50 |  |
| *Selaginella moellendorffii*^2^ | SM00001G06660 |  |
|  | SM00005G01030 |  |
|  | SM00001G06690 |  |
|  | SM00006G06000 |  |
|  | SM00006G05960 |  |
|  | SM00017G01110 |  |
|  | SM00000G03850 |  |
|  | SM00000G03870 |  |
|  | SM00040G00730 |  |
|  | SM00045G00110 |  |
|  | SM00074G00530 |  |
|  | SM00000G05980 |  |
|  | SM00008G04120 |  |
|  | SM00001G06620 |  |
|  | SM00018G03690 |  |
|  | SM00034G00870 |  |
|  | SM00000G03890 |  |
|  | SM00018G03950 |  |
|  | SM00000G03650 |  |
|  | SM00039G01020 |  |
|  | SM00120G00230 |  |
|  | SM00120G00250 |  |
|  | SM00120G00270 |  |
|  | SM00020G01620 |  |
|  | SM00020G01820 |  |
|  | SM00020G01750 |  |
|  | SM00086G00040 |  |
|  | SM00020G01780 |  |
|  | SM00020G01790 |  |
|  | SM00026G01790 |  |
|  | SM00034G02090 |  |
|  | SM00024G01040 |  |
|  | SM00010G04290 |  |
| *Pinus taeda*^3^ | PITA_000000444-RA |  |
|  | PITA_000032443-RA |  |
|  | PITA_000002810-RA |  |
|  | PITA_000053860-RA |  |
|  | PITA_000081231-RA |  |
|  | PITA_000089064-RA |  |
|  | PITA_000089092-RA |  |
|  | PITA_000068640-RA |  |
|  | PITA_000075921-RA |  |
|  | PITAhm_000380-RA |  |
|  | PITA_000018643-RA |  |
|  | PITA_000057755-RA |  |
|  | PITA_000037544-RA |  |
|  | PITA_000081290-RA |  |
|  | PITAhm_002515-RA |  |
|  | PITA_000087462-RA |  |
|  | PITA_000052334-RA |  |
|  | PITA_000018774-RA |  |
|  | PITA_000090552-RA |  |
|  | PITA_000003415-RA |  |
|  | PITAhm_000547-RA |  |
|  | PITA_000064284-RA |  |
|  | PITA_000074983-RA |  |
|  | PITA_000085933-RA |  |
|  | PITA_000075965-RA |  |
|  | PITA_000087551-RA |  |
|  | PITA_000089087-RA |  |
|  | PITA_000090155-RA |  |
|  | PITA_000002172-RA |  |
|  | PITA_000002173-RA |  |
|  | PITA_000034703-RA |  |
|  | PITA_000020970-RA |  |
|  | PITA_000078715-RA |  |
|  | PITA_000093604-RA |  |
|  | PITA_000080767-RA |  |
|  | PITA_000081120-RA |  |
|  | PITA_000059870-RA |  |
|  | PITA_000080108-RA |  |
|  | PITA_000064385-RA |  |
|  | PITA_000090556-RA |  |
|  | PITA_000059893-RA |  |
|  | PITA_000060338-RA |  |
|  | PITA_000046233-RA |  |
|  | PITAhm_001074-RA |  |
|  | PITA_000077719-RA |  |
|  | PITA_000020636-RA |  |
|  | PITA_000079854-RA |  |
|  | PITA_000038511-RA |  |
|  | PITA_000086597-RA |  |
|  | PITA_000040152-RA |  |
|  | PITA_000086089-RA |  |
|  | PITA_000012766-RA |  |
|  | PITA_000073505-RA |  |
|  | PITA_000001529-RA |  |
|  | PITA_000045870-RA |  |
|  | PITA_000053802-RA |  |
|  | PITA_000061394-RA |  |
|  | PITA_000052332-RA |  |
|  | PITA_000072088-RA |  |
|  | PITA_000096332-RA |  |
|  | PITA_000053280-RA |  |
|  | PITA_000018694-RA |  |
|  | PITA_000051560-RA |  |
|  | PITA_000055269-RA |  |
|  | PITA_000027826-RA |  |
|  | PITA_000053187-RA |  |
|  | PITA_000009688-RA |  |
|  | PITA_000021130-RA |  |
|  | PITA_000033455-RA |  |
|  | PITA_000021128-RA |  |
|  | PITA_000022845-RA |  |
|  | PITA_000000451-RA |  |
|  | PITAhm_000685-RA |  |
|  | PITA_000009657-RA |  |
|  | PITA_000054694-RA |  |
|  | PITA_000071802-RA |  |
|  | PITA_000070697-RA |  |
|  | PITA_000060070-RA |  |
|  | PITA_000026353-RA |  |
|  | PITA_000004052-RA |  |
|  | PITA_000020917-RA |  |
|  | PITA_000060878-RA |  |
|  | PITA_000026247-RA |  |
|  | PITA_000073888-RA |  |
|  | PITA_000080668-RA |  |
|  | PITA_000059316-RA |  |
|  | PITA_000087508-RA |  |
|  | PITA_000067795-RA |  |
|  | PITA_000053256-RA |  |
|  | PITA_000057896-RA |  |
|  | PITA_000070115-RA |  |
|  | PITA_000000642-RA |  |
| *Eucalyptus grandis*^4^ | EgranTPS001 |  |
|  | EgranTPS042 |  |
|  | EgranTPS002 |  |
|  | EgranTPS007 |  |
|  | EgranTPS006 |  |
|  | EgranTPS005 |  |
|  | EgranTPS008 |  |
|  | EgranTPS010 |  |
|  | EgranTPS009 |  |
|  | EgranTPS034 |  |
|  | EgranTPS043 |  |
|  | EgranTPS036 |  |
|  | EgranTPS037 |  |
|  | EgranTPS023 |  |
|  | EgranTPS045 |  |
|  | EgranTPS031 |  |
|  | EgranTPS032 |  |
|  | EgranTPS048 |  |
|  | EgranTPS052 |  |
|  | EgranTPS030 |  |
|  | EgranTPS003 |  |
|  | EgranTPS004 |  |
|  | EgranTPS020 |  |
|  | EgranTPS025 |  |
|  | EgranTPS027 |  |
|  | EgranTPS041 |  |
|  | EgranTPS049 |  |
|  | EgranTPS024 |  |
|  | EgranTPS047 |  |
|  | EgranTPS026 |  |
|  | EgranTPS022 |  |
|  | EgranTPS035 |  |
|  | EgranTPS028 |  |
|  | EgranTPS029 |  |
|  | EgranTPS044 |  |
|  | EgranTPS017 |  |
|  | EgranTPS016 |  |
|  | EgranTPS018 |  |
|  | EgranTPS019 |  |
|  | EgranTPS011 |  |
|  | EgranTPS050 |  |
|  | EgranTPS012 |  |
|  | EgranTPS013 |  |
|  | EgranTPS015 |  |
|  | EgranTPS014 |  |
|  | EgranTPS021 |  |
|  | EgranTPS051 |  |
|  | EgranTPS046 |  |
|  | EgranTPS039 |  |
|  | EgranTPS040 |  |
|  | EgranTPS038 |  |
|  | EgranTPS033 |  |
|  | EgranTPS055 |  |
|  | EgranTPS058 |  |
|  | EgranTPS053 |  |
|  | EgranTPS085 |  |
|  | EgranTPS054 |  |
|  | EgranTPS056 |  |
|  | EgranTPS057 |  |
|  | EgranTPS060 |  |
|  | EgranTPS086 |  |
|  | EgranTPS063 |  |
|  | EgranTPS062 |  |
|  | EgranTPS059 |  |
|  | EgranTPS061 |  |
|  | EgranTPS064 |  |
|  | EgranTPS074 |  |
|  | EgranTPS075 |  |
|  | EgranTPS073 |  |
|  | EgranTPS065 |  |
|  | EgranTPS067 |  |
|  | EgranTPS068 |  |
|  | EgranTPS070 |  |
|  | EgranTPS088 |  |
|  | EgranTPS069 |  |
|  | EgranTPS071 |  |
|  | EgranTPS072 |  |
|  | EgranTPS087 |  |
|  | EgranTPS077 |  |
|  | EgranTPS079 |  |
|  | EgranTPS076 |  |
|  | EgranTPS078 |  |
|  | EgranTPS080 |  |
|  | EgranTPS082 |  |
|  | EgranTPS081 |  |
|  | EgranTPS083 |  |
|  | EgranTPS084 |  |
|  | EgranTPS103 |  |
|  | EgranTPS113 |  |
|  | EgranTPS110 |  |
|  | EgranTPS111 |  |
|  | EgranTPS106 |  |
|  | EgranTPS105 |  |
|  | EgranTPS109 |  |
|  | EgranTPS108 |  |
|  | EgranTPS107 |  |
|  | EgranTPS112 |  |
|  | EgranTPS104 |  |
|  | EgranTPS102 |  |
|  | EgranTPS091 |  |
|  | EgranTPS093 |  |
|  | EgranTPS095 |  |
|  | EgranTPS098 |  |
|  | EgranTPS097 |  |
|  | EgranTPS096 |  |
|  | EgranTPS100 |  |
|  | EgranTPS099 |  |
|  | EgranTPS094 |  |
|  | EgranTPS089 |  |
|  | EgranTPS090 |  |
|  | EgranTPS092 |  |
| *Abies grandis*^5^ | AAF61453 | β-phellandrene synthase |
|  | AAB71084 | myrcene synthase |
|  | AAF61455 | α-pinene/limonene synthase |
|  | AAF61454 | terpinolene synthase |
|  | AAB70907 | (--)-limonene synthase |
|  | AAB71085 | (--)-pinene synthase |
|  | AAB70707 | (--)-camphene synthase |
|  | AAC05727 | δ-selinene synthase |
|  | AAC05728 | γ-humulene synthase |
|  | AAC24192 | E-α-bisabolene synthase |
|  | AAB05407 | abietadiene synthase |
| *Picea abies*^5^ | AY473624 | (--)-limonene synthase |
|  | AY473623 | (--)-linalool synthase |
|  | AY473622 | (-)-α/β-pinene synthase |
|  | AY473626 | myrcene synthase |
|  | AY473627 | E,E-α-farnesene synthase |
|  | AY473625 | longifolene synthase |
|  | AY473619 | E-α-bisabolene synthase |
|  | AY473621 | levopimaradiene/abietadiene synthase |
|  | AY473620 | isopimara-7,15-diene synthase |
| *Pieca sitchensis*^5^ | AAP72020 | (--)-pinene synthase |
| *Pinus taeda*^5^ | AAO61228 | (+)-α-pinene synthase |
|  | AAO61225 | (--)-α-pinene synthase |
|  | AAO61227 | α-terpineol synthase |
|  | AAO61226 | α-farnesene synthase |
| *Taxus brevifolia*^5^ | AAC49310 | taxadiene synthase |
| *Ginkgo biloba*^5^ | AAL09965 | levopimaradiene synthase |

^1^ All sequences generated in the present study; ^2^ Sequences retrieved from the PLAZA 2.5 database; ^3^ Sequences retrieved from loblolly pine genome sequencing project [1]; ^4^ Sequences exploited in the study on *Eucalyptus* terpene synthase gene family [2]; ^5^ Sequences exploited in the study on plant terpene synthase gene family [3].

1. Neale D B, Wegrzyn J L, Stevens K A, Zimin A V, Puiu D, Crepeau M W, et al. Decoding the massive genome of loblolly pine using haploid DNA and novel assembly strategies. Genome biology. 2014;15(3): R59.

2. Külheim C, Padovan A, Hefer C, Krause S T, Köllner T G, Myburg A A, et al. The Eucalyptus terpene synthase gene family. BMC genomics. 2015;16(1): 450.

3. Chen F, Tholl D, Bohlmann J, Pichersky E. The family of terpene synthases in plants: a mid-size family of genes for specialized metabolism that is highly diversified throughout the kingdom. The Plant Journal. 2011;66(1): 212-229.
